# Nutritional Characterization of Street Food in Urban Turkmenistan, Central Asia

**DOI:** 10.3389/fpubh.2022.877906

**Published:** 2022-05-23

**Authors:** Gabriela Albuquerque, Sofia Sousa, Inês Lança de Morais, Marcello Gelormini, Carla Motta, Gerard Bryan Gonzales, Azat Ovezov, Albertino Damasceno, Pedro Moreira, João Breda, Nuno Lunet, Patrícia Padrão

**Affiliations:** ^1^EPIUnit, Instituto de Saúde Pública, Porto, Portugal; ^2^Laboratório para a Investigação Integrativa e Translacional em Saúde Populacional (ITR), Porto, Portugal; ^3^Faculdade de Ciências da Nutrição e Alimentação, Universidade do Porto, Porto, Portugal; ^4^Nutrition, Physical Activity and Obesity Programme, Division of Noncommunicable Diseases and Life-Course, World Health Organization Regional Office for Europe, Copenhagen, Denmark; ^5^Instituto Nacional de Saúde Doutor Ricardo Jorge (INSA), Lisboa, Portugal; ^6^Division of Human Nutrition and Health, Wageningen University, Wageningen, Netherlands; ^7^National Public Health and Nutrition Centre, Ashgabat, Turkmenistan; ^8^Departamento de Ciências da Saúde Pública e Forenses e Educação Médica, Faculdade de Medicina da Universidade do Porto, Faculdade de Medicina, Universidade do Porto, Porto, Portugal; ^9^Faculty of Medicine, Eduardo Mondlane University, Maputo, Mozambique; ^10^Centro de Atividade Física, Saúde e Lazer, Universidade do Porto, Porto, Portugal; ^11^WHO Regional Office for Europe, Athens, Greece

**Keywords:** Central Asia, food processing, nutritional value, ready-prepared foods, street food, Turkmenistan

## Abstract

**Objective:**

Describing the availability and nutritional composition of the most commonly available street foods in *Ashgabat*, Turkmenistan.

**Methods:**

One hundred sixty-one street food vending sites (six public markets) were assessed, through a collection of data on vending sites' characteristics and food availability, and samples of commonly available foods (21 homemade; 11 industrial), for chemical analysis.

**Results:**

Fruit, beverages, and food other than fruit were available in 6.8, 29.2, and 91.9% of all vending sites, respectively. Regarding the latter, 52.7% of the vending sites sold only homemade products (main dishes, snacks, cakes, biscuits and pastries, bread, ice-cream chocolate and confectionery, savory pastries and sandwiches), 37.2% only industrial (ice-cream, chocolate and confectionery, cakes, biscuits and pastries, snacks, bread and savory pastries) and 10.1% both. Homemade foods presented significantly higher total fat [homemade 11.6 g (range 6.6–19.4 g); industrial 6.2 g (range 4.0–8.6 g), *p* = 0.001], monounsaturated, polyunsaturated and trans-fat, and sodium and potassium content per serving. Industrial wafers presented the highest mean saturated (11.8 g/serving) and trans-fat (2.32 g/serving) content. Homemade hamburgers presented the highest mean sodium content (1889 mg/serving).

**Conclusions:**

Strategies to encourage the production and sales of healthier street foods, especially homemade, are needed to promote healthier urban food environments in urban Turkmenistan.

## Introduction

Non-communicable diseases (NCDs) are the leading cause of death and disability-adjusted life-years globally and in the World Health Organization (WHO) European region, where they account for 70% of all deaths (1). A more burdensome scenario is observed in Central Asian countries, such as Turkmenistan, where NCDs account for 76% of all deaths. Of these, cardiovascular diseases and cancer are among the leading causes of mortality (47.0 and 11.0%, respectively). Moreover, the high prevalence of obesity (18% in adults), diabetes, and other NCDs coexist with micronutrient deficiencies (2); high blood pressure, dietary risks, high body mass index, tobacco, use and malnutrition have been identified as important risk factors for disease burden in Turkmenistan (3). These observations seem to be in line with the reported double burden of malnutrition and nutrition transition in Central Asia (4).

The nutrition transition in the region has been characterized by a decreased consumption of cereal, roots, and tubers, and an increased availability of animal products, sugar, sweeteners, and vegetable oils in recent decades (5). It has also been observed a transition from traditional to more globalized eating patterns. Socioeconomic changes in the region, such as urbanization and increased participation of women in the urban working force, are leading to an increased consumption of food away from home (6), such as street food.

Street food is very popular worldwide, especially in low and middle-income countries (LMIC), due to its low-cost, convenience, considerable portion sizes and taste (7, 8). It has been described that it often replaces home meals, while also being a significant contributor to excess intake of energy and nutrients in several urban settings worldwide (8). Street food is part of the Central Asian gastronomy (9), reflecting locally available food products, practices and consumer preferences (10, 11). Nevertheless, the types of items available and their nutritional composition (8), as well as the role of these smaller businesses in the local food environments remain understudied, particularly in the region (8, 12, 13).

Turkmenistan is an upper-middle-income country in Central Asia, which has experienced positive economic development since the early 2000s, with the annual GDP growth being 6.2% in 2016. The population of the country has also been increasing in this period and was about 5.6 million people in 2016, of which 50.7% lived in urban areas (14). The capital city, *Ashgabat*, is the largest in the country, housing approximately 8,10,000 inhabitants (2018 data) (15). Furthermore, this is one of the countries in the WHO European region where a lack of representative surveys on nutritional status, dietary habits and food composition has been identified (16). Taking all this into consideration, the aim of this study was to characterize the street food environment in *Ashgabat*, focusing on food availability and the nutritional composition of the most commonly available foods and beverages.

## Methods

### Setting, Study Design and Eligibility Criteria

This cross-sectional study was implemented within the scope of the FEEDcities project, supported by the WHO – Europe, and followed a stepwise standardized methodology (based on primary data collection through observation of vending sites and interviews with the vendors and chemical analysis of food samples) to characterize the urban food environment in countries in Central Asia and Eastern Europe (17). An evaluation, conducted in October 2016, was designed to provide an overall description of the urban food environment in *Ashgabat*, Turkmenistan [namely including fast-food vending sites, supermarkets and street food vending sites (18)]. This study assesses, specifically, the availability and nutritional composition of street food. The street food definition adopted in this study was the one proposed by Food and Agriculture Organization and WHO, as “ready-to-eat foods and beverages prepared and/or sold by vendors or hawkers especially in the streets and other similar places” (19, 20).

Information provided by local authorities and gathered during field visits before study implementation outlined that most street food vending sites were concentrated in traditional outdoor markets and their surroundings. Thus, local authorities selected six markets, from a total of 10 identified in *Ashgabat*, where this study would be conducted. To define the study area, a 500-m buffer was built around the centroid of each of the selected markets, covering the markets and their surroundings.

Eligible vending sites were defined as the business establishments selling ready-to-eat food, including beverages and/or snacks, from any venue other than permanent storefront businesses or establishments with four permanent walls not selling directly to the street, operating in the predefined perimeter. This included mobile vendors, as well as sellers with semi-static or stationary vending units. The exclusion criteria were the following: (1) food establishments with four permanent walls; (2) permanent storefront business; (3) street vendors selling exclusively non-food products or raw foods not ready-to-eat; (4) food stalls and carts that were part of permanent stores or licensed establishments.

### Data Collection: Vending Sites, Vendors and Food Availability

The markets were assessed in seven consecutive days. Field researchers, operating in pairs, canvassed systematically each study area to find street food vendors. The vendors from all eligible vending sites were invited to participate.

At each vending site, after registering the Global Positioning System (GPS) coordinates, the interviewers collected the following information, through direct observation: sex of the vendor, mobility of the vending site and type of physical setup. Stationary vending sites were further classified into formal [stand, showcase, kiosk, cafeteria (vending site with no waiter service, where food is displayed in aligned counters/stalls or booths and customers take their desired food as they walk along)] or improvised (bench with table, vending machine, and other improvised sites such as popcorn machines, refrigerators or freezer machine selling soft ice-cream).

The vendor was then approached by the interviewers, who explained the study objectives and procedures and asked for verbal informed consent to participate in the study. When the vendor agreed, the interviewers carried out computer-assisted personal interviewing, enquiring about food availability, including serving sizes (18). All the 161 eligible street food vendors approached agreed to participate.

Foods available were grouped according to their nature, into fruit (product in *natura*, either fresh or dry), beverages (any alcoholic and non-alcoholic drink) or food other than fruit. Food other than fruit was further classified as homemade (foods of domestic manufacture cooked and/or prepared at home or on the street, even if using industrial ingredients) or industrial (food products produced by the food industry and sold as is without further preparation and/or cooking). Homemade food was also grouped according to the preparation method in cooked or uncooked. Both homemade and industrial foods were grouped according to broader food groups, that were created based on the groups of the WHO nutrient profile model (21): (1) bread; (2) cakes, biscuits and pastries; (3) main dishes (4) sandwiches; (5) savory pastries, (6) snacks and (7) ice-cream, chocolate and confectionery. Beverages were further classified into soft drinks, water, fruit juice-based drinks, fresh fruit juice-based drinks, milk, energy drinks, coffee, tea, fruit smoothies (ice and natural fruit extract-based beverages), alcoholic and traditional beverages. The latter included *ayran* (savory yogurt-based beverage, made by mixing yogurt with water and salt), kephyr (fermented milk drink made with a yeast or bacterial fermentation starter of kefir grains), *yogurt and kompot* (made by cooking fruit in a large volume of water, often with sugar or raisins), non-alcoholic.

### Food Sample Collection

Following the computation of the frequency of each of the identified foods and beverages across all the vending sites included in the study, samples of the most commonly available foods and beverages were collected for nutritional composition assessment. The most frequent homemade foods (*n* = 21) were bread (namely *chiorek* and *milk chiorek*), sandwiches and main dishes (doner kebab, fried potatoes, hamburger, hot-dog, pizza, cabbage salad and lentil soup), savory pastries (baked or fried; filled with meat, vegetables or potato, such as sausage roll, *samsa, pirozhki, fitchi, pirog* and *chebureki*), a snack (boiled corn cob) and cakes and pastries (biscuits, bun, muffin (*keksi*),cake and wafers). The most frequent industrial foods (*n* = 11) were bread, snacks (croutons, chips, salty sticks), biscuits, muffin (*keksi*), gingerbread biscuit (*pryaniki*), wafers, halva, chocolate and ice-cream.

The selection of the vending sites where the food samples were collected was carried out randomly, from the list of the GPS coordinates of the eligible vending sites previously assessed. A sample of each food product, corresponding to one serving, was bought whenever possible at these vending sites. If it was not possible to buy the target foods at the selected coordinates, a systematic selection procedure was followed, in which field researchers start moving north from that point and change direction clockwise (first east, then south, then west, then north again) whenever the limits of the study area or a physical barrier (such as a wall or a canal) were met until reaching vending sites where these foods were available (18). In each vending site, only one sample was collected.

For each selected homemade and industrial food, a total of four samples was defined to be collected from different vending sites. Nevertheless, it was not possible to achieve this for most of them (*n* = 28, out of 32). A total of 76 samples (55 homemade and 21 industrial) were collected in four consecutive days.

### Nutritional Composition Assessment

After collection, samples were homogenized, weighted and stored in a freezer (−18°C) until the nutritional composition assessment. Total fat determination was performed according to AOAC 948.15, 2000, methodology with an acid hydrolysis method followed by Soxhlet extraction with petroleum ether (40 – 60°C) as the extraction solvent (22).

Fatty acid profile was determined by gas chromatography. Analysis was performed according to the ISO 12966 (2015–2017) and the Commission Regulation (EC) No. 796/2002 (2002), with slight modifications, as described by Albuquerque et al. (23). A gas chromatograph (Hewlett Packard 7890B series GC-Systems, Waldbronn, Germany) equipped with a flame ionization detector (FID) and a 100 m, 0.25 mm ID, 0.2um column (Agilent HP-88, Santa Clara, United States) was used. Helium was used as a carrier gas at a flow rate of 1.0 ml/min. The oven temperature was programmed as follows: 50°C for 1 min, then to 175°C at 10°C/min, held for 15 min, then finally to 240°C at 4°C/min, held for 15 min. The injector and detector temperatures were 280 and 280°C, respectively. The identification of the fatty acid methyl esters was based on comparison of the retention times of sample peaks with those of a commercially available FAME mixture (Supelco 37 Component FAME Mix) from the supplier (Sigma-Aldrich Co. LLC). The results were expressed on a relative fatty acids basis and grouped as saturated (SFA), monounsaturated (MUFA), polyunsaturated (PUFA), n-3 and n-6 fatty and trans fatty acids (TFA).

Sodium and potassium analyses were performed after an acid digestion in a closed-vessel microwave system (ETHOS UP Microwave Digestion System), as described by Nascimento et al. (24), followed by determination of minerals using an inductively coupled plasma optical emission spectrometer (Agilent 5110 ICP-OES). The analytical results were the average of two determinations per food sample. If the coefficient of variation of duplicates was above 5% for fatty acids and micronutrients another two replicates where performed. In this case, the average of two determinations consistent with the acceptance criterion was calculated. All the analytical results were expressed by serving size, in g.

### Statistical Analysis

The vending sites and food availability were characterized through absolute and relative frequencies (categorical variables). Pearson's χ2 test was used to identify statistically significant differences between formal and informal street food vending sites. Markets were defined as the sampling units. The statistical analyses were conducted adjusting for the clustering at the sampling unit level.

Regarding the nutritional composition assessment, mean serving sizes per food, in g, were calculated as the mean weight of the individual samples collected for each of the foods. Likewise, per-serving levels of each nutrient were calculated as the mean content of the individual samples and expressed in g/serving (macronutrients) or mg/serving (micronutrients). Results were presented for each food, as the mean and range of total fat, including SFA, MUFA, PUFA, n-3 fatty acids, n-6 fatty acids, TFA, sodium and potassium per serving and molar sodium-to-potassium (Na/K) ratios. Contents of sodium and potassium of each sample were converted to millimoles using their molar weights, 23.0 g/mol and 39.1 g/mol, respectively, to calculate individual molar Na/K ratios. The nutritional composition of homemade and industrial foods was compared using the nonparametric Mann–Whitney U test.

A *p*-value (*p*) < 0.05 was considered statistically significant. Statistical analysis was performed using the software STATA® version 15.1 for Windows®.

## Results

### Food Vending Sites and Vendors

Supplementary Figure 1 depicts the distribution of the selected markets throughout *Ashgabat* city. All vending sites were stationary, of which the most frequent physical setups were formal, such as *stand* (45.6%) and showcases (23.9%). Nearly three in every four street food vendors were women (68.9%).

### Food Availability

Fruit, beverages and food other than fruit were available, respectively, in 6.8, 29.2 and 91.9% of the vending sites. Over half of the vending sites (52.7%) sold only homemade, 37.2% only industrial and 10.1% both homemade and industrial foods. Among the vending sites selling homemade foods, all had cooked foods available, while 35.5% of them sold prepared foods. Main dishes, snacks and cakes, biscuits and pastries were the most commonly available groups of homemade foods, in at least 20% of these vending sites. Ice-cream, chocolate and confectionery, cakes, cookies and sweet pastries and snacks were the most frequent groups of industrial foods, available in over 50% of the stationary vending sites selling industrial foods. The most commonly available beverages were soft drinks (78.7%), water (76.6%) and traditional beverages (39.7%). Overall, there were no statistically significant differences in street food availability between improvised and formal street food outlets. The only exceptions were prepared homemade foods, which were more frequent in formal street food vending sites (improvised: *n* = 3; 18.8%, formal: *n* = 30; 39.0%, *p* = 0.005), and homemade snacks (improvised: *n* = 11; 68.8%, formal: *n* = 15; 19.5%, *p* = 0.007), industrial cakes, biscuits and pastries (improvised: *n* = 5; 100.0%, formal: *n* = 42; 64.6%, *p* = 0.013), and tea (improvised: *n* = 3; 37.5%, formal: *n* = 5; 12.8%, *p* = 0.044), which were more frequently available in improvised street food vending sites (Table 1).

**Table 1:** Food offer by type of vending site in Ashgabat, Turkmenistan.

	**Total (*****n*** **=** **161)**		**Type of vending site**				
			**Improvised (*****n*** **=** **21)**		**Formal (*****n*** **=** **140)**		***p***
	***n***	**%**	***n***	**%**	***n***	**%**	
Fruit	11	6.8	2	9.5	9	6.4	0.508
Food other than fruit	148	91.9	20	95.2	128	91.4	0.668
Industrial	55	37.2	4	20.0	51	39.8	0.208
Homemade and industrial	15	10.1	1	5.0	14	10.9	
Homemade	78	52.7	15	75.0	63	49.2	
Homemade foods: preparation^a^							
Cooked	93	100.0	16	100.0	57	74.0	0.074
Prepared but non-cooked	33	35.5	3	18.8	30	39.0	0.005^e^
Homemade foods: groups^b^							
Main dishes	33	35.5	3	18.8	30	39.0	0.124
Snacks	26	28.0	11	68.8	15	19.5	0.007^e^
Cakes, biscuits and pastries	19	20.4	0	0.0	19	24.7	0.083
Bread	18	19.4	0	0.0	18	23.4	0.122
Ice-cream, chocolate and confectionery	16	17.2	2	12.5	14	18.2	0.549
Savoury pastries	13	14.0	2	12.5	11	14.3	0.694
Sandwiches	12	12.9	3	18.8	9	11.7	0.528
Industrial foods: groups^c^							
Ice-cream, chocolate and confectionery	54	77.1	3	60.0	51	78.5	0.632
Cakes, biscuits and pastries	47	67.1	5	100.0	42	64.6	0.013^e^
Snacks	39	55.7	2	40.0	37	56.9	0.712
Bread	14	20.0	0	0.0	14	21.5	0.209
Savoury pastries	2	2.9	0	0.0	2	3.1	0.426
Beverages	47	29.2	8	38.1	39	27.9	0.202
Soft drinks	37	78.7	8	100.0	29	74.4	0.157
Water	36	76.6	8	100.0	28	71.8	0.110
Traditional beverages^d^	27	39.7	6	37.5	21	40.4	0.431
Fruit juice-based drinks	22	46.8	5	62.5	17	43.6	0.437
Tea	8	17.0	3	37.5	5	12.8	0.044^e^
Coffee	5	10.6	2	25.0	3	7.7	0.110
Fresh Fruit juice-based drinks	5	10.6	0	0.0	5	12.8	0.589
Milk	4	8.5	0	0.0	4	10.3	0.245
Alcoholic beverages	4	8.5	0	0.0	4	10.3	0.263
Energy drinks	3	6.4	2	25.0	1	2.6	0.116
Fruit smoothies	1	2.1	0	0.0	1	2.6	0.725

a *The sum of the values for this variable is higher than the total number of homemade foods, as each vendor could offer foods prepared in different ways*.

b *The sum of the values for this variable is higher than the total number of homemade foods, as each vendor could offer foods from different groups*.

c *The sum of the values for this variable is higher than the total number of industrial foods, as each vendor could offer foods from different groups*.

d *Traditional beverages (non-alcoholic): yoghurt (n = 12), ayran (n = 9), kephyr (n = 5), and kompot (n = 1)*.

e *Statistically significant differences according to Pearson's Chi-square test, for a confidence level of 95% (p-value < 0.05)*.

### Nutritional Composition

Overall, homemade foods presented significantly higher total fat (median g/serving: 11.6 vs. 6.2, *p* = 0.001), MUFA (median g/serving: 3.4 vs. 1.9, *p* < 0.001), PUFA (median g/serving: 2.7 vs. 1.0, *p* < 0.001), TFA (median g/serving: 0.2 vs. 0.0, *p* = 0.020), sodium (median mg/serving: 573 vs. 99, *p* < 0.001) and potassium (median mg/serving: 219 vs. 104, *p* < 0.001) contents per serving than industrial foods. In contrast, industrial foods presented, per 100g, a significantly higher content of total fat (median g/100 g: 17.1 vs. 8.8, *p* < 0.001), MUFA (median g/100 g: 5.2 vs. 2.3, *p* < 0.001) and potassium (median mg/100 g: 236 vs. 172, *p* < 0.001). These foods also presented higher SFA content per serving (median: 43.1 vs. 34.1, *p* = 0.014), and per 100 g (median: 2.8 vs. 7.1, *p* < 0.001). The nutritional composition of the collected street foods, per 100 g, is presented in Supplementary Tables 2, 3.

Homemade hamburger and fried potatoes presented the highest mean total fat (32.1 and 32.8 g/serving, respectively). Hamburger presented, in addition, the highest mean MUFA content (10.1 g/serving), while fried potatoes presented, in addition, among the highest mean PUFA content (13.0 g/serving), particularly *n*−6 (12.6 g/serving) and n-3 (0.5 g/serving), although the highest PUFA and n-6 contents were found in homemade *pirozhky* (18.5 and 18.4 g/serving, respectively). Homemade cake and soup presented among the highest values of n-3 fatty acids (0.2 g/serving). Homemade hamburger (11.1 g/serving) and industrial wafer (11.8 g/serving) presented the highest SFA contents. The highest mean TFA contents were found in homemade and industrial wafers (respectively, 0.65 and 1.81 g/serving) (Table 2).

**Table 2:** Nutritional composition (Total fat, MUFA, PUFA, SFA and TFA), per serving, of the street food samples collected in Ashgabat.

	***N***	**Mean serving size (min-max)** **(g/serving)**		**Mean total fat (min-max)** **(g/serving)**		**Mean MUFA (min-max)** **(g/serving)**		**Mean PUFA (min-max)** **(g/serving)**		**Mean** ***n*****−6 (min-max)** **(g/serving)**		**Mean** ***n*****−3 (min-max)** **(g/serving)**		**Mean SFA (min-max)** **(g/serving)**		**Mean TFA (min-max)** **(g/serving)**	
**Industrial**																
Biscuits	3	30	(29–31)	5.6	(4.4–7.3)	1.4	(0.7–2.2)	0.6	(0.3–1.2)	0.6	(0.3–1.2)	0.0	(0.0–0.1)	3.3	(2.7–3.7)	0.07	(0.01–0.20)
Bread	2	50	(50–50)	1.6	(1.1–2.1)	0.4	(0.2–0.5)	0.8	(0.6–1.0)	0.8	(0.6–1.0)	0.0	(0.0–0.0)	0.3	(0.1–0.5)	0.01	(0.01–0.01)
Chips	2	20	(20–20)	6.0	(5.0–7.0)	1.5	(1.4–1.6)	0.4	(0.3–0.4)	0.4	(0.3–0.4)	0.0	(0.0–0.0)	3.8	(3.0–4.5)	0.06	(0.02–0.09)
Chocolate	1	33	(33–33)	7.4	(7.4–7.4)	0.6	(0.6–0.6)	0.3	(0.3–0.3)	0.3	(0.3–0.3)	0.0	(0.0–0.0)	6.1	(6.1–6.1)	0.01	(0.01–0.01)
Croutons	1	38	(38–38)	4.6	(4.6–4.6)	2.0	(2.0–2.0)	0.7	(0.7–0.7)	0.6	(0.6–0.6)	0.0	(0.0–0.0)	1.5	(1.5–1.5)	0.18	(0.18–0.18)
*Halva*	2	20	(20–20)	8.0	(8.0–8.1)	2.3	(2.2–2.4)	4.0	(4.0–4.0)	4.0	(4.0–4.0)	0.0	(0.0–0.1)	1.4	(1.3–1.4)	0.01	(0.00–0.01)
Ice-cream	1	70	(70–70)	4.6	(4.6–4.6)	1.4	(1.4–1.4)	0.4	(0.4–0.4)	0.4	(0.4–0.4)	0.0	(0.0–0.0)	2.5	(2.5–2.5)	0.01	(0.01–0.01)
*Keksi* (muffin)	2	49	(49–49)	9.0	(8.5–9.6)	2.9	(2.5–3.3)	0.8	(0.6–0.9)	0.7	(0.6–0.8)	0.0	(0.0–0.1)	4.6	(3.4–5.7)	0.39	(0.26–0.52)
*Pryaniki*	2	60	(55–64)	3.1	(2.6–3.5)	0.9	(0.8–1.0)	1.4	(1.3–1.4)	1.4	(1.3–1.4)	0.0	(0.0–0.0)	0.6	(0.2–1.1)	0.01	(0.01–0.01)
Salty sticks	2	48	(46–50)	8.7	(7.7–9.7)	2.8	(2.4–3.2)	1.9	(1.1–2.6)	1.8	(0.9–2.6)	0.1	(0.0–0.1)	3.6	(2.2–5.0)	0.09	(0.04–0.14)
Wafers	3	94	(71–108)	26.2	(16.2–35.7)	9.8	(5.6–14.3)	1.6	(0.9–2.9)	1.5	(0.8–2.7)	0.1	(0.0–0.1)	11.8	(8.4–17.2)	1.81	(0.45–4.45)
**Homemade**																	
Biscuits	2	33	(32–33)	8.5	(7.8–9.2)	3.0	(2.5–3.4)	1.1	(0.9–1.3)	1.0	(0.8–1.2)	0.1	(0.1–0.2)	3.3	(1.9–4.6)	0.76	(0.33–1.19)
Boiled corn	3	152	(132–163)	1.9	(0.0–3.2)	0.5	(0.0–0.8)	0.9	(0.0–1.4)	0.8	(0.0–1.3)	0.1	(0.0–0.1)	0.4	(0.0–0.8)	0.01	(0.00–0.03)
Bread (*chiorek*)	4	120	(120–120)	2.0	(1.3–3.9)	0.3	(0.2–0.5)	1.3	(0.8–2.5)	1.2	(0.8–2.4)	0.1	(0.0–0.2)	0.3	(0.2–0.6)	0.01	(0.00–0.02)
Bread (*milk chorek*)	3	120	(120–120)	1.1	(0.7–1.5)	0.2	(0.1–0.3)	0.6	(0.4–0.8)	0.5	(0.3–0.7)	0.1	(0.0–0.1)	0.2	(0.2–0.3)	0.00	(0.00–0.00)
Bun	3	94	(71–119)	2.4	(1.1–4.1)	0.6	(0.4–1.0)	0.8	(0.4–1.2)	0.7	(0.4–1.2)	0.0	(0.0–0.0)	0.9	(0.2–1.7)	0.02	(0.01–0.03)
*Chebureki*	4	99	(47–128)	9.3	(3.0–15.2)	2.1	(0.7–3.8)	4.2	(1.6–6.1)	4.0	(1.5–5.6)	0.1	(0.0–0.5)	2.5	(0.5–5.9)	0.13	(0.04–0.20)
*Doner kebab*	3	260	(249–270)	28.0	(17.1–37.9)	8.0	(5.2–11.9)	8.0	(5.5–13.0)	7.9	(5.4–12.8)	0.1	(0.1–0.2)	10.1	(5.4–17.5)	0.54	(0.21–0.13)
*Fitchi*	3	192	(164–243)	13.0	(8.4–15.6)	4.4	(2.7–5.4)	2.2	(1.9–2.7)	2.1	(1.7–2.7)	0.1	(0.0–0.2)	5.4	(3.4–6.8)	0.46	(0.07–0.86)
Fried potatoes	3	195	(175–206)	32.8	(11.2–69.1)	7.9	(1.3–12.6)	13.0	(5.1–27.8)	12.6	(3.8–27.8)	0.5	(0.0–1.3)	10.1	(0.8–25.2)	0.31	(0.05–0.48)
Hamburger	1	288	(288–288)	32.1	(32.1–32.1)	10.1	(10.1–10.1)	9.3	(9.3–9.3)	9.1	(9.1–9.1)	0.1	(0.1–0.1)	11.1	(11.1–11.1)	0.17	(0.17–0.17)
Hot-dog	3	217	(143–346)	17.0	(6.8–31.0)	4.5	(1.8–6.8)	7.9	(1.9–17.2	7.8	(1.9–17.2)	0.1	(0.0–0.2)	3.7	(2.8–5.5)	0.14	(0.03–0.25)
*Keksi* (muffin)	2	101	(86–115)	15.4	(7.6–23.3)	4.6	(3.5–5.6)	5.6	(0.9–10.4)	5.5	(0.8–10.3)	0.1	(0.1–0.1)	4.3	(2.7–5.9)	0.29	(0.20–0.39)
*Pirog* (savoury pie)	4	158	(108–213)	12.2	(1.4–20.9)	4.0	(0.5–6.7)	2.0	(0.2–4.2)	2.0	(0.2–4.2)	0.0	(0.0–0.1)	5.5	(0.5–9.9)	0.18	(0.02–0.35)
*Pirozhky*	4	100	(91–121)	30.3	(5.1–95.4)	5.5	(1.1–16.8)	18.5	(2.7–62.7)	18.4	(2.7–62.4)	0.1	(0.0–0.3)	4.7	(1.0–11.0)	0.26	(0.03–0.73)
*Pirozhnoe* (cake)	2	102	(91–113)	17.7	(15.2–20.1)	5.3	(4.5–6.0)	3.7	(1.3–6.1)	3.6	(1.2–5.9)	0.2	(0.0–0.4)	7.6	(6.5–8.6)	0.38	(0.15–0.60)
Pizza	1	174	(174–174)	13.2	(13.2–13.2)	3.3	(3.3–3.3)	2.6	(2.6–2.6)	2.5	(2.5–2.5)	0.0	(0.0–0.0)	6.5	(6.5–6.5)	0.21	(0.21–0.21)
Salad (cabbage)	2	101	(99–104)	9.1	(8.1–10.0)	1.9	(1.8–2.0)	4.0	(3.9–4.0)	3.9	(3.9–3.9)	0.0	(0.0–0.1)	2.6	(1.7–3.6)	0.14	(0.07–0.22)
*Samsa*	1	110	(110–110)	7.9	(7.9–7.9)	2.5	(2.5–2.5)	0.6	(0.6–0.6)	0.6	(0.6–0.6)	0.0	(0.0–0.0)	4.2	(4.2–4.2)	0.13	(0.13–0.13)
Sausage roll	3	84	(71–98)	5.9	(4.8–6.5)	1.4	(1.1–1.9)	2.6	(1.8–3.8)	2.6	(1.7–3.8)	0.0	(0.0–0.0)	1.5	(1.1–2.1)	0.07	(0.01–0.19)
Soup (lentil)	1	382	(382–382)	15.7	(15.7–15.7)	6.6	(6.6–6.6)	3.7	(3.7–3.7)	3.6	(3.6–3.6)	0.2	(0.2–0.2)	4.4	(4.4–4.4)	0.39	(0.39–0.39)
Wafers	3	88	(69–102)	19.9	(8.1–30.1)	5.8	(2.5–8.4)	3.4	(1.5–6.7)	3.4	(1.5–6.6)	0.0	(0.0–0.1)	9.2	(3.7–17.3)	0.65	(0.04–1.09)

*SFA, saturated fatty acids; MUFA, monounsaturated fatty acids; PUFA, polyunsaturated fatty acids; TFA, trans fatty acids*.

The mean sodium content ranged between 9 mg/serving in industrial halva and 1,889 mg/serving in homemade hamburger whereas mean potassium content ranged between 33 mg/serving in homemade biscuits and 1,299 mg/serving in homemade fried potatoes. From the 32 foods analyzed, only six presented average sodium/potassium ratio below 1: industrial chocolate and halva (0.2), wafers (0.3), homemade boiled corn and fried potatoes (0.4), industrial ice-cream (0.9), homemade ice-cream (0.5) and chocolate (0.6). The highest sodium/potassium ratio was found in industrial salty sticks (17.3) (Table 3).

**Table 3:** Nutritional composition (Na, K and Na/K), per serving, of the street food samples collected in Ashgabat.

	**N**	**Mean serving size (min-max)** **(g/serving)**		**Mean Na (min-max)** **(mg/serving)**		**Mean K (min-max)** **(mg/serving)**		**Mean Na/K (min-max)**	
**Industrial**					
Biscuits	3	30	(29–31)	66	(44–87)	68	(44–100)	1.9	(0.7–2.5)
Bread	2	50	(50–50)	272	(222–323)	114	(103–125)	4.2	(3.0–5.3)
Chips	2	20	(20–20)	129	(74–184)	89	(29–149)	5.7	(0.8–10.7)
Chocolate	1	33	(33–33)	30	(30–30)	246	(246–246)	0.2	(0.2–0.2)
Croutons	1	38	(38–38)	161	(161–161)	81	(81–81)	3.4	(3.4–3.4)
Halva	2	20	(20–20)	9	(9–10)	99	(96–102)	0.2	(0.2–0.2)
Ice–cream	1	70	(70–70)	39	(39–39)	71	(71–71)	0.9	(0.9–0.9)
*Keksi* (muffin)	2	49	(49–49)	153	(128–178)	115	(101–129)	2.2	(2.2–2.3)
*Pryaniki*	2	60	(55–64)	63	(57–68)	103	(92–114)	1.0	(1.0–1.1)
Salty sticks	2	48	(46–50)	885	(739–1031)	95	(60–130)	17.3	(13.5–2.1)
Wafers	3	94	(71–108)	93	(83–111)	520	(362–725)	0.3	(0.2–0.4)
**Homemade**									
Biscuits	2	33	(32–33)	112	(86–137)	33	(32–33)	5.8	(4.4–7.2)
Boiled corn	3	152	(132–163)	80	(21–195)	401	(326–475)	0.4	(0.1–1.0)
Bread (*chiorek*)	4	120	(120–120)	560	(411–713)	180	(149–211)	5.2	(4.7–5.7)
Bread (milk *chorek*)	3	120	(120–120)	667	(582–767)	179	(164–202)	6.3	(6.0–6.5)
Bun	3	94	(71–119)	121	(79–203)	118	(90–163)	1.6	(1.3–2.1)
*Chebureki*	4	99	(47–128)	487	(267–650)	177	(146–232)	4.6	(3.1–6.0)
*Doner kebab*	3	260	(249–270)	985	(799–1228)	711	(570–927)	2.4	(1.7–3.3)
*Fitchi*	3	192	(164–243)	1157	(756–1470)	313	(242–447)	6.5	(5.2–8.7)
Fried potatoes	3	195	(175–206)	342	(135–568)	1299	(1175–1504)	0.4	(0.2–0.6)
Hamburger	1	288	(288–288)	1889	(1889–1889)	620	(620–620)	5.2	(5.2–5.2)
Hot–dog	3	217	(143–346)	1005	(902–1169)	588	(255–1252)	4.6	(1.6–6.2)
*Keksi* (muffin)	2	101	(86–115)	356	(355–357)	121	(106–136)	5.1	(4.4–5.7)
*Pirog* (savoury pie)	4	158	(108–213)	983	(541–1454)	263	(169–356)	6.2	(5.4–6.9)
*Pirozhky*	4	100	(91–121)	355	(100–554)	165	(108–206)	3.6	(1.6–5.8)
*Pirozhnoe* (cake)	2	102	(91–113)	277	(258–296)	163	(135–190)	3.0	(2.3–3.7)
Pizza	1	174	(174–174)	1061	(1061–1061)	379	(379–379)	4.7	(4.7–4.7)
Salad (cabbage)	2	101	(99–104)	789	(682–896)	227	(214–239)	5.9	(5.4–6.3)
*Samsa*	1	110	(110–110)	488	(488–488)	144	(144–144)	5.8	(5.8–5.8)
Sausage roll	3	84	(71–98)	377	(209–591)	140	(95–186)	4.7	(3.0–7.2)
Soup (lentil)	1	382	(382–382)	1189	(1189–1189)	695	(695–695)	2.9	(2.9–2.9)
Wafers	3	88	(69–102)	112	(52–168)	114	(40–181)	1.8	(1.6–2.2)

*K, potassium; Na, sodium; Na/K sodium-potassium ratio*.

## Discussion

In *Ashgabat*, street food vending sites selling homemade street foods were the most frequent. Main dishes, snacks and cakes, biscuits and sweet pastries were the most commonly available groups of homemade foods, while ice-cream, chocolate and confectionery, cakes, biscuits and pastries and snacks were the most commonly available groups of industrial foods. The homemade foods presented higher fat (including MUFA, PUFA and TFA), sodium and potassium content per serving than industrial foods, while the later were richer in total fat, MUFA, SFA and potassium per 100 g.

The higher availability of homemade cooked foods may corroborate previous evidence that street food usually replaces home-cooked meals (8). Notwithstanding, when analyzing the absolute frequency of homemade and industrial food groups in this street food environment, industrial snacks, cakes, biscuits and savory pastries are the ones available in a larger number of vending sites in comparison with, for example, homemade main dishes, the most popular homemade foods group. This, aligned with the observation that homemade snacks, cakes, biscuits and sweet pastries were also common, might indicate a predominance of snacking options among street foods in *Ashgabat*, both industrial and homemade. Data retrieved from the Food Systems Dashboard shows, in line with these findings, a recent trend of increased availability of packaged and ultra-processed foods at the national level (25). In this study, it was also observed a coexistence of both westernized and traditional options among the most commonly available beverages (e.g. soft drinks and traditional drinks) and foods other than fruit (e.g. hamburger, fried potatoes and *doner kebab*, lentil soup). Altogether, these findings might suggest a possible westernization of food habits in urban Turkmenistan, as observed in other Central Asian urban centers (26, 27), with traditional foods and beverages becoming replaced by westernized options and, eventually, main meals by snacking meals.

Homemade street foods are usually available in large servings, being one of the reasons for their popularity (7, 8), a fact also observed in this study. Another difference was additionally observed, regarding the nature of the predominant homemade and industrial food groups, with main dishes and sandwiches (served in larger servings) being only available as homemade options. This may contribute to justify the discrepancies found in the comparison of the nutritional composition per serving and per 100 grams of food. The highest mean fat content values per serving found in homemade main dishes, sandwiches and savory pastries (such as hamburger, *doner kebab*, fried potatoes and *pirozhky*) and in industrial wafers are also in line with previous findings among street foods in Central Asia (26, 27) and Eastern Europe (28). The contribution of each fatty acid to total fat varied widely by food group. The SFA content exceeded 40% of total fat in homemade main dishes and savory pastries, in homemade and industrial bakery products, as well as industrial confectionery and snacks. The mean TFA contribution for the total fat was particularly high in homemade cakes and pastries, reaching a maximum of approximately 10% in biscuits, which is concerning given that TFA were proven to be exceptionally harmful even in small intake amounts (2% of total energy intake) (29, 30). Although there is a dearth of data regarding nutritional composition of food in Turkmenistan, similar TFA levels were reported in industrial biscuits, cakes and wafers commercialized in 2015–16 in countries of the former Soviet Union (31). The heterogeneous fatty acid profile found might reflect not only the wide variability between food groups, as also within the same group, including differences in food preparation. For example, the most complex preparations such as main dishes and sandwiches might involve the use of different cooking methods, and varieties and quantities of ingredients, specifically fats and oils (8). A study conducted in urban and rural slums in India reported an association between the high SFA and TFA amounts in savory pastries and snacks and the oils used to cook them (32). In addition, shortenings, also rich in these fatty acids, are still traditionally used in bakery cooking as they provide desirable tenderness, texture and extend a product's shelf life at a low cost (33, 34). Moreover, although the oils and fats used to cook may conform with quality standards, unsafe cooking practices such as continuous reuse of oil for frying may lead to increased TFA concentrations (35) as well as the formation of other toxic compounds.

In this study, traditional homemade foods were the main sources of sodium, with some main dishes, sandwiches and snacks surpassing half the daily recommended intake of 2,000 mg and, for example, hamburger reaching 95% of this recommendation (36). Some homemade street foods, such as bread and lentil soup, deserve particular attention since they might be expected to be examples of healthier street foods, but present considerable amounts of sodium. This might be even more noticeable in the case of bread, which might be consumed several times throughout the day, inclusively during main meals. Similar findings have been described among street foods in Tajikistan and Kyrgyzstan (37) and, altogether, these might suggest alignment with global observations that meat, bread and bakery products are among the main contributors to daily sodium intake (38). Findings from national studies in Turkmenistan, conducted, respectively in 2017 and 2018, highlight a mean daily sodium intake among the adult population of approximately double the recommended [4,400 mg (25) and 3,800 mg (39)].

Most homemade and industrial street foods groups presented mean molar sodium-to-potassium ratios above the optimal ratio of 1, recommended by the WHO to prevent NCDs (36, 40). This underlines the high sodium content of these foods but also highlights their generally low potassium content (only five out of the 32 analyzed street foods complied with this recommendation). Some garnishes and sandwiches, including potato or vegetables, such as fried potatoes and *doner kebab*, had the highest content of this nutrient [representing 23.8–32.5% of the minimum daily intake recommended 3,510 mg (41)], but most foods presented a maximum of 20% of its minimum daily recommended intake. This observation reinforces the need to increase the availability of nutritionally dense foods and ingredients, such as vegetables and fruit, to the urban population of Turkmenistan. Promoting the consumption of potato, a traditional staple, together with vegetables and pulses, could be an interesting and sustainable strategy to increase potassium availability, coupled with an effort to rely on healthier cooking practices than frying, and to decrease the amount of salt and fat added in preparation.

Many characteristics of the street food availability in the urban food environment of *Ashgabat* seem to be in line with the nutrition transition occurring in Central Asia (5, 42, 43), with important health impacts. Regarding its nutritional composition, the high fat (namely SFA and TFA) and sodium content and low potassium content are among the main risk factors for the development of NCDs (42) and may also contribute to justify the high prevalence and mortality by NCDs in the country, particularly cardiovascular disease (2, 3), and the relatively low life expectancy at birth [68.2 years, in 2019 (14)]. From a public health perspective, it seems timely to address these issues in the design and implementation of national and local health policies, following the global recommendation for increasing local-level interventions, in order to tackle health inequities (44). Following the need for increasing awareness of the harmfulness of TFA and sodium, even in small amounts, several international efforts to eliminate industrially-produced TFA (34) and sodium (45) from the global food supply have been recently intensified, especially in the WHO European region. The WHO recommends that total TFA intake should be limited to <1% of total energy intake, corresponding to <2.2 g/day in the diet of a regular adult (2,000 kcal) (30). In Turkmenistan, there are currently no regulations limiting the content of SFA or TFA, neither sodium in foods, in opposition to some of its neighboring countries, members of the Eurasian Economic Union, which have adopted a regulatory limit of 2 g TFA/100 g fat in foods, in partnership between national governments and the food industry (46). Nevertheless, the adoption of similar measures in Turkmenistan might contribute to improving the lipid profile of street food. Despite the challenge of monitoring homemade foods, it is expected that the industrial ingredients used in their preparation would have to follow these standards, resulting, thus in similar compliance to that of industrial foods. In sum, the findings of this study highlight a need to study the commonly used ingredients and practices in street food preparation and the street food manufacturers' motivations to do so. The study of customers' motivations for buying street food and their nutritional literacy could additionally contribute to the design of nutritional education strategies specific to the needs and preferences of all the involved stakeholders.

This study might contribute to reinforce previous literature on street food, providing insight into an understudied region (12) and more urbanized settings (28). The stepwise approach aimed to ensure an unbiased and comprehensive characterization of the street foods available in Ashgabat's foodscape, particularly on the nutritional composition of those most frequent, which reflect their popularity. Although the results may not be generalized due to local cultural specificities, the vast potential of the methodology to be adapted to different settings (17) allows comparison of results. The nutritional composition of the analyzed foods was estimated by chemical analysis, which overcomes limitations of previous studies (8, 12, 42). Future assessments could comprise a broader array of nutrients and energy. In this study, the option for the presentation per serving aimed to emphasize the role of the serving size when discussing the nutritional quality of food. Nevertheless, given that in this research area it is common to present the nutritional composition per 100 g of food, this respective information is available in Supplementary Tables 1, 2. Above all, the work developed under this study is anticipated to have contributed to narrowing the evidence gap on food, nutrition and NCDs in Turkmenistan and Central Asia. The findings have the potential to support current efforts in national health and food systems in the scope of the Decade of Action on Nutrition (2016–2025) (47) and contribute to the evolution of healthcare, health and food policy, ultimately improving the population's health.

## Data Availability Statement

The data analyzed in this study is subject to the following licenses/restrictions. The participants of this study did not agree for their data to be shared publicly, so supporting data is not available. Requests to access these datasets should be directed to GA, gabriela.albuquerque@ispup.up.pt.

## Ethics Statement

The studies involving human participants were reviewed and approved by Ethics Committee of the Institute of Public Health of the University of Porto. Written informed consent for participation was not required for this study in accordance with the national legislation and the institutional requirements.

## Author Contributions

MG, AD, PM, JB, NL, and PP designed the study. IL supervised the study implementation and data collection. CM and GG were responsible for the conception of the methodology, training and supervision of the chemical analysis of the food samples collected, and preliminary analysis of these results. GA and PP performed the analysis and interpretation of the results. GA drafted the manuscript. All authors critically revised the manuscript and gave their final approval of the manuscript submitted for publication.

## Funding

The FEEDcities project was funded by the World Health Organization Europe (WHO registration 2015/591370 and 2017/698514). This study was financed through national funding from the Foundation for Science and Technology—FCT (Portuguese Ministry of Science, Technology and Higher Education), under the project UIDB/04750/2020. Individual PhD grants attributed to GA (SFRH/BD/118630/2016) and SS (SFRH/BD/130650/2017) were funded by FCT and the Programa Operacional Capital Humano (POCH/FSE). The funders had no role in the design, analysis or writing of this paper.

## Conflict of Interest

The authors declare that the research was conducted in the absence of any commercial or financial relationships that could be construed as a potential conflict of interest.

## Publisher's Note

All claims expressed in this article are solely those of the authors and do not necessarily represent those of their affiliated organizations, or those of the publisher, the editors and the reviewers. Any product that may be evaluated in this article, or claim that may be made by its manufacturer, is not guaranteed or endorsed by the publisher.
