# Supplementary figures and images for: Nutritional Characterization of Street Food in Urban Turkmenistan, Central Asia
Source: Front Public Health. 2022 May 23;10:877906. doi: 10.3389/fpubh.2022.877906 (PMC9168320; doi:10.3389/fpubh.2022.877906)

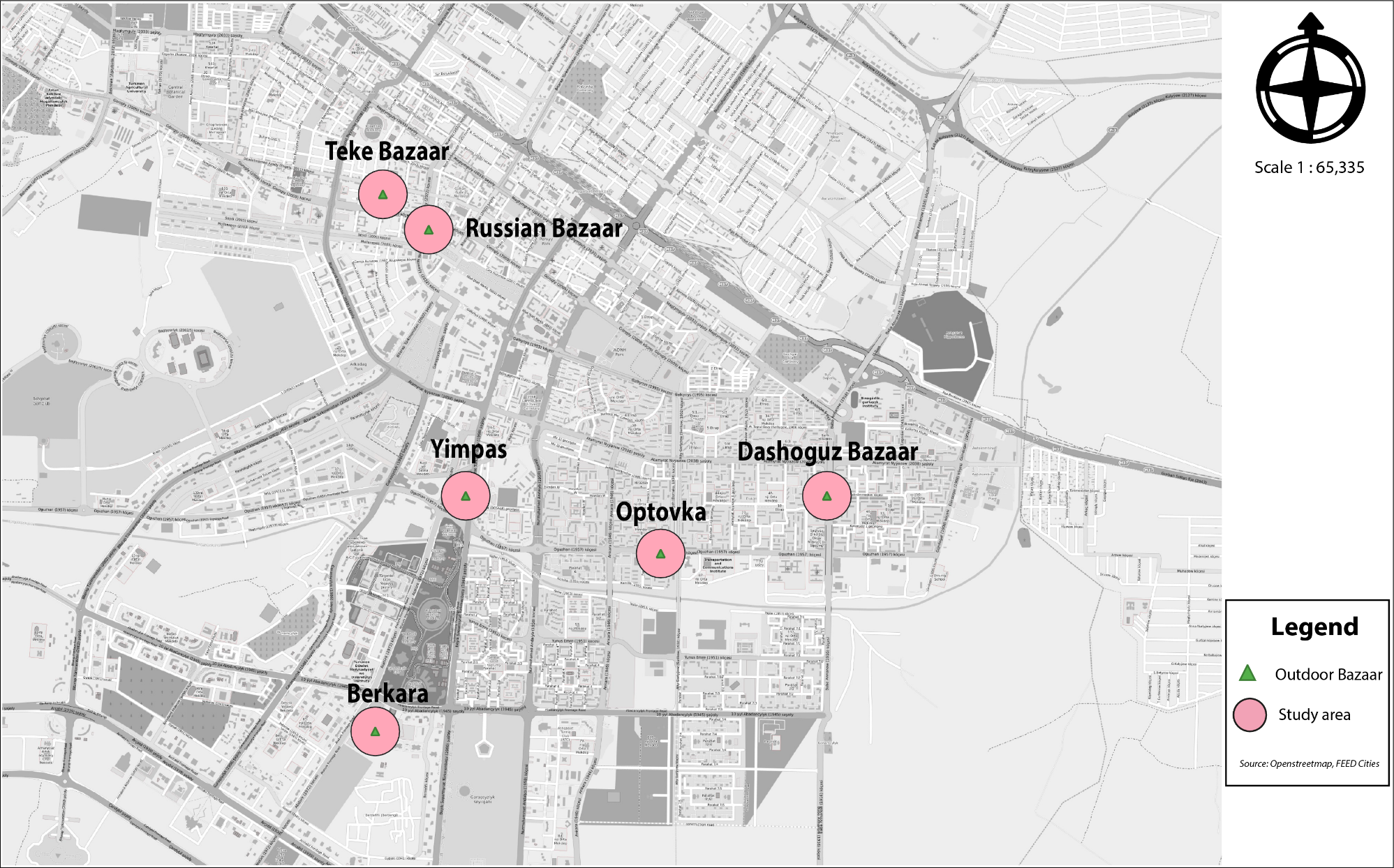


**Supplementary figure 1. Geographical distribution of the markets in Ashgabat, Turkmenistan.**

Supplement: Supplementary file 1 [file Table_1.DOCX]
