# Supplementary material for: Nutritional Characterization of Street Food in Urban Turkmenistan, Central Asia
Source: Front Public Health. 2022 May 23;10:877906. doi: 10.3389/fpubh.2022.877906 (PMC9168320; doi:10.3389/fpubh.2022.877906)
Supplement: Supplementary file 2 [file Table_2.DOCX]

| **Supplementary Table 1. Examples of street food samples collected in Ashgabat** | |
| --- | --- |
| **Industrial** | |
| *Halva*: sweet traditional dessert, crumbly and usually made from honey or sugar, butter and [*tahini*](https://en.wikipedia.org/wiki/Tahini) ([sesame](https://en.wikipedia.org/wiki/Sesame) paste) or other types of [butter](https://en.wikipedia.org/wiki/Nut_butter), such as [sunflower](https://en.wikipedia.org/wiki/Sunflower)  butter. It may also contain nuts, such as walnuts, sunflower seeds, almonds, sesame seeds, peanuts | 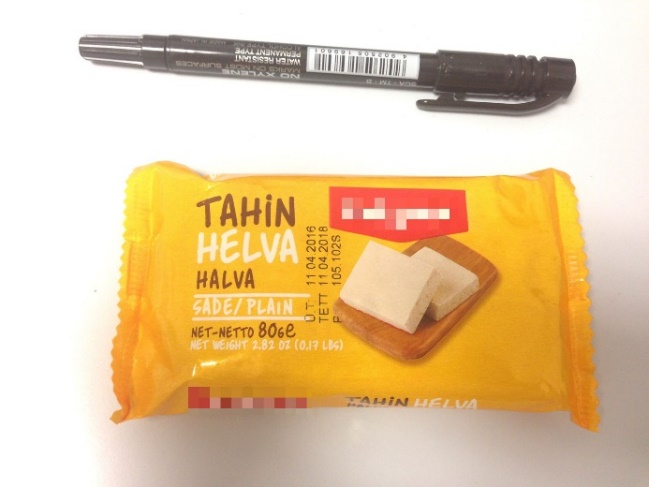 |
| *Pryaniki:* gingerbread biscuit | 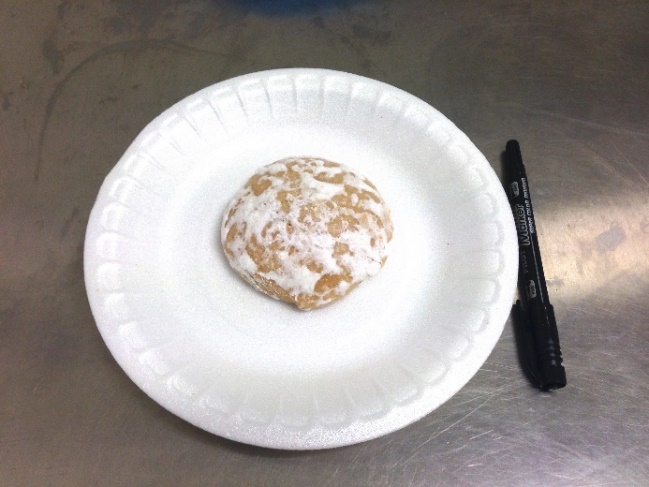 |
| **Homemade** | |
| Bread *(chorek):* A flat bread, made of flour, salt, yeast and butter. | 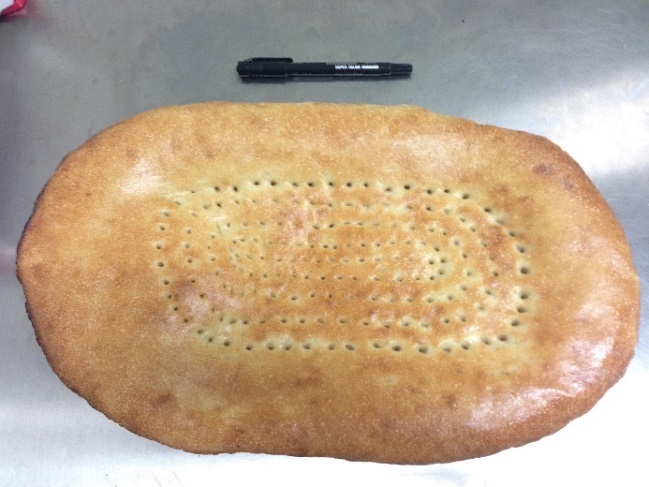 |
| Bread *(milk chorek):* flatbread whose ingredients are similar to traditional *chorek*, with the addition of milk. | 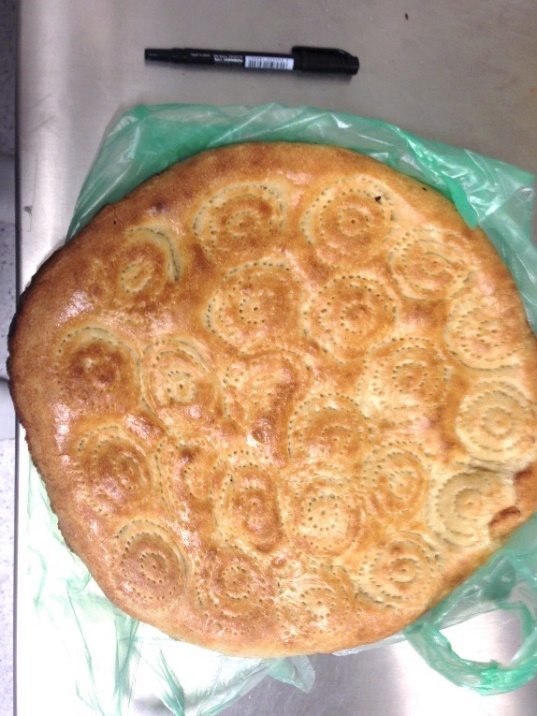 |
| Bun | 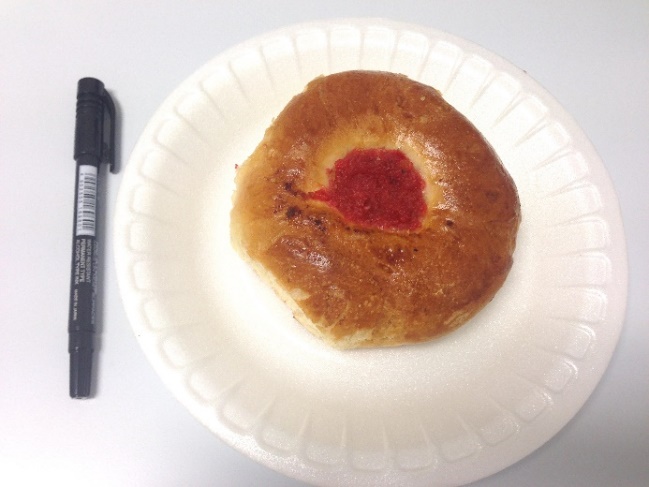 |
| *Cheburek:* fried savoury pastry generally filled with ground or minced meat and onions | 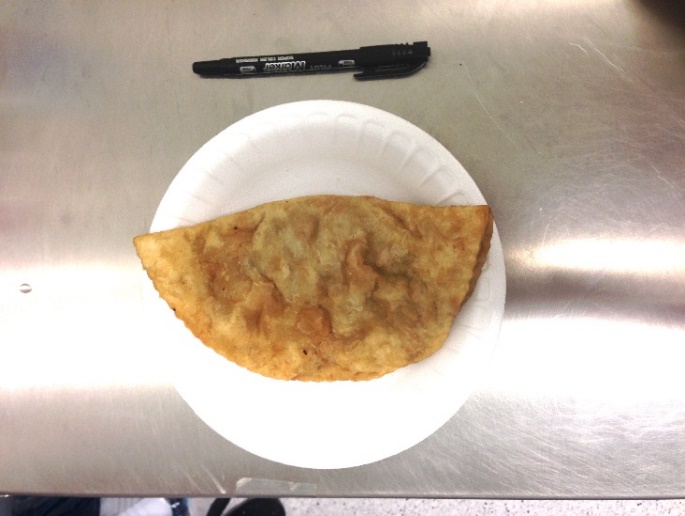 |
| *Doner kebab:* seasoned meat stacked in the shape of an inverted cone turned slowly in front of a heat source | 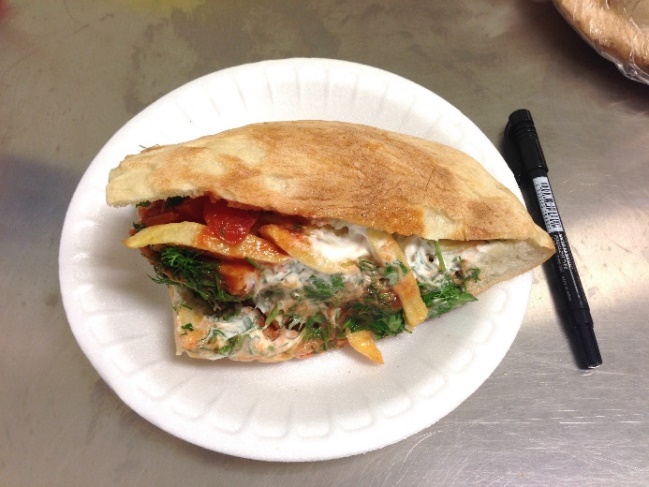 |
| *Fitchi:* traditional meat pie, usually beef or lamb | 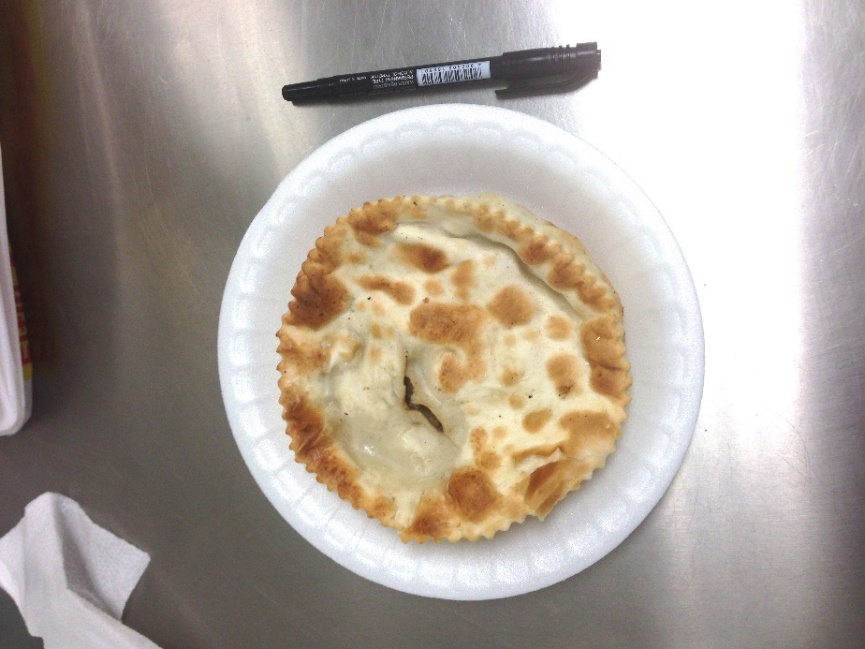 |
| *Pirog* (savoury pie): baked yeast pastry commonly stuffed with meat (typically beef or mutton) and/or vegetables, usually served in a pie shape | 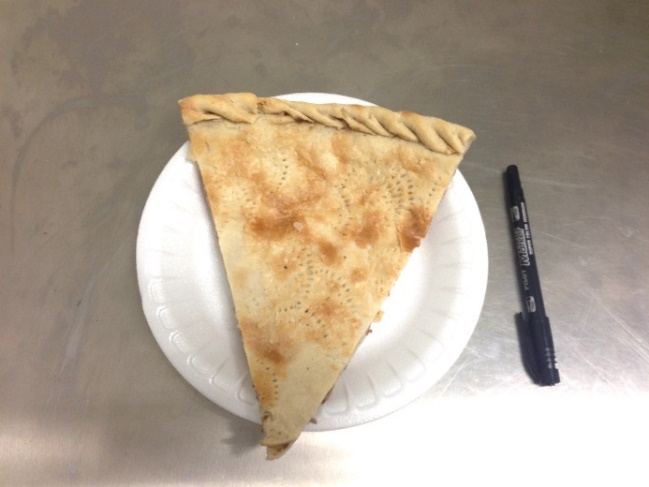 |
| *Piroshky:* baked or fried yeast pastry commonly stuffed with meat (typically beef or mutton) and/or vegetables, usually served in small portions | 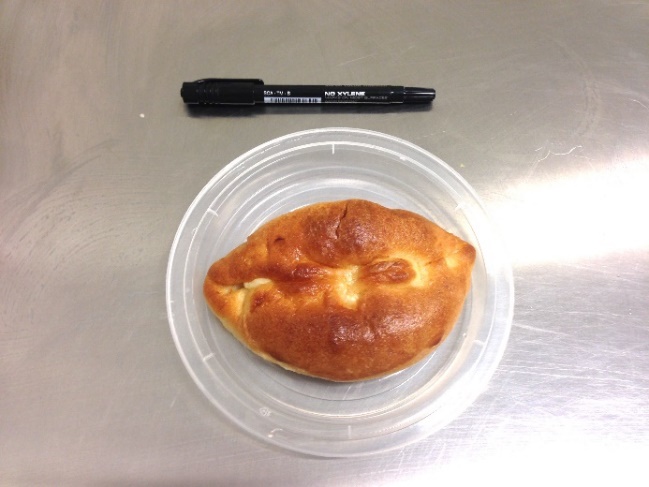 |
| *Pirozhnoe:* cake | 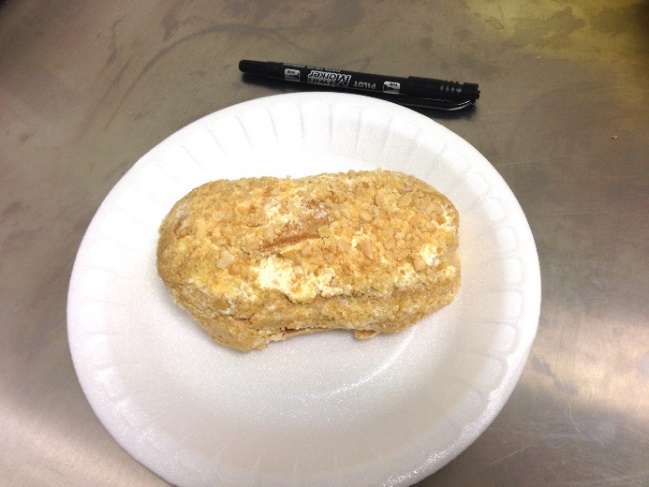 |
| *Somsa:* baked puff pastry usually filled with ground meat (lamb, beef or chicken) and vegetables | 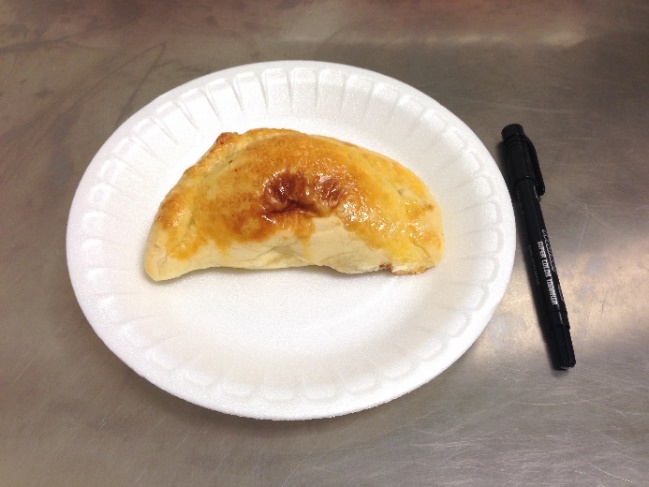 |

| **Supplementary Table 2. Nutritional composition (Total fat, MUFA, PUFA, SFA and TFA), per 100g, of the street food samples collected in Ashgabat.** | | | | | | | | | | | | | | | | | |
| --- | --- | --- | --- | --- | --- | --- | --- | --- | --- | --- | --- | --- | --- | --- | --- | --- | --- |
|  | **N** | **Mean serving size (min-max)**  **(g/100 g)** | | **Mean total fat (min-max)**  **(g/100 g)** | | **Mean MUFA(min-max)**  **(g/100 g)** | | **Mean PUFA(min-max)**  **(g/100 g)** | | **Mean n-6(min-max)**  **(g/100 g)** | | **Mean n-3 (min-max)**  **(g/100 g)** | | **Mean SFA (min-max)**  **(g/100 g)** | | **Mean TFA (min-max)**  **(g/100 g)** | |
|  |  |  |  |  |  |  |  |  |  |  |  |  |  |  |  |  |  |
| **Industrial** |  |  | |  |  |  |  |  |  |  |  |  |  |  |  |  |  |
| **Biscuits** | 3 | 30 | (29-31) | 5.6 | (4.4-7.3) | 1.4 | (0.7-2.2) | 0.6 | (0.3-1.2) | 0.6 | (0.3-1.2) | 0.0 | (0.0-0.1) | 3.3 | (2.7-3.7) | 0.07 | (0.01-0.20) |
| **Bread** | 2 | 50 | (50-50) | 1.6 | (1.1-2.1) | 0.4 | (0.2-0.5) | 0.8 | (0.6-1.0) | 0.8 | (0.6-1.0) | 0.0 | (0.0-0.0) | 0.3 | (0.1-0.5) | 0.01 | (0.01-0.01) |
| **Chips** | 2 | 20 | (20-20) | 6.0 | (5.0-7.0) | 1.5 | (1.4-1.6) | 0.4 | (0.3-0.4) | 0.4 | (0.3-0.4) | 0.0 | (0.0-0.0) | 3.8 | (3.0-4.5) | 0.06 | (0.02-0.09) |
| **Chocolate** | 1 | 33 | (33-33) | 7.4 | (7.4-7.4) | 0.6 | (0.6-0.6) | 0.3 | (0.3-0.3) | 0.3 | (0.3-0.3) | 0.0 | (0.0-0.0) | 6.1 | (6.1-6.1) | 0.01 | (0.01-0.01) |
| **Croutons** | 1 | 38 | (38-38) | 4.6 | (4.6-4.6) | 2.0 | (2.0-2.0) | 0.7 | (0.7-0.7) | 0.6 | (0.7-0.7) | 0.0 | (0.0-0.0) | 1.5 | (1.5-1.5) | 0.18 | (0.18-0.18) |
| ***Halva*** | 2 | 20 | (20-20) | 8.0 | (8.0-8.1) | 2.3 | (2.2-2.4) | 4.0 | (4.0-4.0) | 4.0 | (4.0-4.0) | 0.0 | (0.0-0.1) | 1.4 | (1.3-1.4) | 0.01 | (0.00-0.01) |
| **Ice-cream** | 1 | 70 | (70-70) | 4.6 | (4.6-4.6) | 1.4 | (1.4-1.4) | 0.5 | (0.5-0.5) | 0.4 | (0.5-0.5) | 0.0 | (0.0-0.0) | 2.5 | (2.5-2.5) | 0.01 | (0.01-0.01) |
| ***Keksi* (muffin)** | 2 | 49 | (49-49) | 9.1 | (8.5-9.6) | 2.9 | (2.5-3.3) | 0.8 | (0.6-0.9) | 0.7 | (0.6-0.9) | 0.0 | (0.0-0.1) | 4.6 | (3.4-5.7) | 0.39 | (0.26-0.52) |
| ***Pryaniki*** | 2 | 60 | (55-64) | 3.1 | (2.6-3.5) | 0.9 | (0.8-1) | 1.4 | (1.3-1.4) | 1.4 | (1.3-1.4) | 0.0 | (0.0-0.0) | 0.6 | (0.2-1.1) | 0.01 | (0.01-0.01) |
| **Salty sticks** | 2 | 48 | (46-50) | 8.7 | (7.6-9.7) | 2.8 | (2.4-3.2) | 1.9 | (1.1-2.6) | 1.8 | (1.1-2.6) | 0.1 | (0.0-0.1) | 3.6 | (2.2-5.0) | 0.05 | (0.04-0.14) |
| **Wafers** | 3 | 94 | (71-108) | 26.2 | (16.2-35.7) | 9.8 | (5.6-14.3) | 1.6 | (0.9-2.9) | 1.5 | (0.9-2.9) | 0.1 | (0.0-0.1) | 11.8 | (8.4-17.2) | 1.81 | (0.45-4.45) |
| **Homemade** |  |  |  |  |  |  |  |  |  |  |  |  |  |  |  |  |  |
| **Biscuits** | 2 | 33 | (32-33) | 8.5 | (7.8-9.2) | 3.0 | (2.5-3.4) | 1.1 | (0.9-1.3) | 1.0 | (0.9-1.3) | 0.1 | (0.1-0.2) | 3.3 | (1.9-4.6) | 0.76 | (0.33-1.19) |
| **Boiled corn** | 3 | 152 | (132-163) | 1.9 | (0.0-3.2) | 0.5 | (0.0-0.8) | 0.9 | (0.0-1.4) | 0.8 | (0.0-1.4) | 0.1 | (0.0-0.1) | 0.4 | (0.0-0.8) | 0.01 | (0.00-0.03) |
| **Bread (*chiorek*)** | 4 | 120 | (120-120) | 2.0 | (1.3-3.9) | 0.3 | (0.2-0.5) | 1.3 | (0.8-2.6) | 1.2 | (0.8-2.6) | 0.1 | (0.0-0.2) | 0.3 | (0.2-0.6) | 0.01 | (0.00-0.02) |
| **Bread (*milk* *chorek*)** | 3 | 120 | (120-120) | 1.1 | (0.7-1.5) | 0.2 | (0.1-0.3) | 0.6 | (0.4-0.8) | 0.5 | (0.4-0.8) | 0.1 | (0.0-0.1) | 0.2 | (0.2-0.3) | 0.00 | (0.00-0.00) |
| **Bun** | 3 | 94 | (71-119) | 2.4 | (1.1-4.1) | 0.6 | (0.4-1.0) | 0.8 | (0.4-1.2) | 0.7 | (0.4-1.2) | 0.0 | (0.0-0.0) | 0.9 | (0.2-1.7) | 0.02 | (0.01-0.03) |
| ***Chebureki*** | 4 | 99 | (47-128) | 9.3 | (3.0-15.2) | 2.1 | (0.1-3.0) | 4.2 | (1.6-6.1) | 4.0 | (1.6-6.1) | 0.1 | (0.0-0.5) | 2.5 | (0.5-5.9) | 0.14 | (0.04-0.20) |
| ***Doner* *kebab*** | 3 | 260 | (249-270) | 28.0 | (17.1-37.9) | 8.0 | (5.2-11.9) | 8.1 | (5.5-13.0) | 7.9 | (5.5-13.0) | 0.1 | (0.1-0.2) | 10.1 | (5.4-17.5) | 0.54 | (0.21-1.13) |
| ***Fitchi*** | 3 | 192 | (164-243) | 13.0 | (8.4-15.6) | 4.4 | (2.7-5.4) | 2.2 | (1.9-2.6) | 2.1 | (1.9-2.6) | 0.1 | (0.0-0.2) | 5.4 | (3.4-6.8) | 0.46 | (0.07-0.86) |
| **Fried potatoes** | 3 | 195 | (175-206) | 32.8 | (11.2-69.1) | 7.9 | (1.3-12.6) | 13.0 | (5.1-27.8) | 12.6 | (5.1-27.8) | 0.5 | (0.0-1.3) | 10.1 | (0.8-25.2) | 0.31 | (0.05-0.48) |
| **Hamburger** | 1 | 288 | (288-288) | 32.1 | (32.1-32.1) | 10.1 | (10.1-10.1) | 9.3 | (9.3-9.3) | 9.2 | (9.3-9.3) | 0.1 | (0.1-0.1) | 11.1 | (11.1-11.1) | 0.17 | (0.17-0.17) |
| **Hot-dog** | 3 | 217 | (143-346) | 17.0 | (6.8-31.0) | 4.5 | (1.8-6.8) | 7.9 | (1.9-17.2) | 7.8 | (1.9-17.2) | 0.1 | (0.0-0.2) | 3.7 | (2.8-5.5) | 0.14 | (0.03-0.25) |
| ***Keksi* (muffin)** | 2 | 101 | (86-115) | 15.4 | (7.6-23.3) | 4.6 | (3.5-5.6) | 5.6 | (0.9-10.3) | 5.5 | (0.9-10.3) | 0.1 | (0.1-0.1) | 4.3 | (2.7-5.9) | 0.30 | (0.20-0.39) |
| ***Pirog* (savoury pie)** | 4 | 158 | (108-213) | 12.2 | (1.4-20.9) | 4.0 | (0.5-6.7) | 2.0 | (0.2-4.2) | 2.0 | (0.2-4.2) | 0.0 | (0.0-0.1) | 5.5 | (0.5-9.9) | 0.18 | (0.02-0.35) |
| ***Pirozhky*** | 4 | 100 | (91-121) | 30.3 | (5.1-95.4) | 5.5 | (1.1-16.8) | 18.5 | (2.7-62.7) | 18.4 | (2.7-62.7) | 0.1 | (0.0-0.3) | 4.7 | (1.0-11.0) | 0.26 | (0.03-0.73) |
| ***Pirozhnoe* (cake)** | 2 | 102 | (91-113) | 17.7 | (15.2-20.0) | 5.3 | (4.5-6.0) | 3.7 | (1.3-6.1) | 3.6 | (1.3-6.1) | 0.2 | (0.0-0.4) | 7.6 | (6.5-8.6) | 0.38 | (0.15-0.60) |
| **Pizza** | 1 | 174 | (174-174) | 13.2 | (13.2-13.2) | 3.3 | (3.3-3.3) | 2.6 | (2.6-2.6) | 2.5 | (2.6-2.6) | 0.0 | (0.0-0.0) | 6.5 | (6.5-6.5) | 0.21 | (0.21-0.21) |
| **Salad (cabbage)** | 2 | 101 | (99-104) | 9.1 | (8.1-10.0) | 1.9 | (1.8-2.0) | 4.0 | (3.9-4.0) | 3.9 | (3.9-4.0) | 0.0 | (0.0-0.1) | 2.6 | (1.7-3.6) | 0.15 | (0.07-0.22) |
| ***Samsa*** | 1 | 110 | (110-110) | 7.9 | (7.9-7.9) | 2.5 | (2.5-2.5) | 0.6 | (0.6-0.6) | 0.6 | (0.6-0.6) | 0.0 | (0.0-0.0) | 4.2 | (4.2-4.2) | 0.13 | (0.13-0.13) |
| **Sausage roll** | 3 | 84 | (71-98) | 5.9 | (4.8-6.5) | 1.4 | (1.1-1.9) | 2.6 | (1.8-3.8) | 2.6 | (1.8-3.8) | 0.0 | (0.0-0.1) | 1.5 | (1.1-2.1) | 0.07 | (0.01-0.19) |
| **Soup (lentil)** | 1 | 382 | (382-382) | 15.7 | (15.7-15.7) | 6.6 | (6.6-6.6) | 3.7 | (3.7-3.7) | 3.6 | (3.7-3.7) | 0.2 | (0.2-0.2) | 4.4 | (4.4-4.4) | 0.39 | (0.39-0.39) |
| **Wafers** | 3 | 88 | (69-102) | 19.9 | (8.1-30.2) | 5.8 | (2.5-8.4) | 3.4 | (1.5-6.7) | 3.4 | (1.5-6.7) | 0.0 | (0.0-0.1) | 9.2 | (3.7-17.3) | 0.65 | (0.04-1.09) |
| SFA, saturated fatty acids. MUFA, monounsaturated fatty acids. PUFA, polyunsaturated fatty acids. TFA, trans fatty acids | | | | | | | | | | | | | | | | | |

| **Supplementary Table 3. Nutritional composition (Na, K and Na/K), per 100g, of the street food samples collected in Ashgabat.** | | | | | | | | | |
| --- | --- | --- | --- | --- | --- | --- | --- | --- | --- |
|  | **N** | **Mean serving size (min-max)**  **(g/100 g)** | | **Mean Na (min-max)**  **(mg/100 g)** | | **Mean K (min-max)**  **(mg/100 g)** | | **Mean Na/K**  **(min-max)** | |
|  |  |  |  |  |  |  |  |  |  |
| **Industrial** |  |  | |  | |  | |  | |
| **Biscuits** | 3 | 30 | (29-31) | 221 | (151-286) | 230 | (151-347) | 1.9 | (0.7-2.5) |
| **Bread** | 2 | 50 | (50-50) | 544 | (443-645) | 228 | (205-251) | 4.2 | (3.0-5.3) |
| **Chips** | 2 | 20 | (20-20) | 645 | (369-922) | 446 | (147-746) | 5.7 | (0.8-10.7) |
| **Chocolate** | 1 | 33 | (33-33) | 90 | (90-90) | 743 | (743-743) | 0.2 | (0.2-0.2) |
| **Croutons** | 1 | 38 | (38-38) | 423 | (423-423) | 212 | (212-212) | 3.4 | (3.4-3.4) |
| **Halva** | 2 | 20 | (20-20) | 46 | (42-50) | 494 | (478-509) | 0.2 | (0.2-0.2) |
| **Ice-cream** | 1 | 70 | (70-70) | 56 | (56-56) | 102 | (102-102) | 0.9 | (0.9-0.9) |
| ***Keksi* (muffin)** | 2 | 49 | (49-49) | 315 | (263-367) | 236 | (206-266) | 2.2 | (2.2-2.3) |
| ***Pryaniki*** | 2 | 60 | (55-64) | 105 | (105-105) | 173 | (167-178) | 1.0 | (1.0-1.1) |
| **Salty sticks** | 2 | 48 | (46-50) | 1834 | (1619-2049) | 194 | (131-258) | 17.3 | (13.5-2.1) |
| **Wafers** | 3 | 94 | (71-108) | 101 | (81-120) | 549 | (437-703) | 0.3 | (0.2-0.4) |
| **Homemade** |  |  |  |  |  |  |  |  |  |
| **Biscuits** | 2 | 33 | (32-33) | 341 | (270-412) | 101 | (97-104) | 5.8 | (4.4-7.2) |
| **Boiled corn** | 3 | 152 | (132-163) | 58 | (13-147) | 263 | (246-297) | 0.4 | (0.1-1.0) |
| **Bread (*chiorek*)** | 4 | 120 | (120-120) | 467 | (343-595) | 150 | (125-176) | 5.2 | (4.7-5.7) |
| **Bread (milk *chorek*)** | 3 | 120 | (120-120) | 556 | (485-639) | 149 | (137-168) | 6.3 | (6.0-6.5) |
| **Bun** | 3 | 94 | (71-119) | 123 | (88-170) | 125 | (98-140) | 1.6 | (1.3-2.1) |
| ***Chebureki*** | 4 | 99 | (47-128) | 503 | (405-582) | 197 | (132-311) | 4.6 | (3.1-6.0) |
| ***Doner* *kebab*** | 3 | 260 | (249-270) | 379 | (322-471) | 272 | (229-344) | 2.4 | (1.7-3.3) |
| ***Fitchi*** | 3 | 192 | (164-243) | 603 | (462-741) | 160 | (144-184) | 6.5 | (5.2-8.7) |
| **Fried potatoes** | 3 | 195 | (175-206) | 171 | (77-279) | 668 | (592-740) | 0.4 | (0.2-0.6) |
| **Hamburger** | 1 | 288 | (288-288) | 657 | (657-657) | 215 | (215-215) | 5.2 | (5.2-5.2) |
| **Hot-dog** | 3 | 217 | (143-346) | 517 | (338-659) | 233 | (156-362) | 4.6 | (1.6-6.2) |
| ***Keksi* (muffin)** | 2 | 101 | (86-115) | 361 | (307-414) | 120 | (117-123) | 5.1 | (4.4-5.7) |
| ***Pirog* (savoury pie)** | 4 | 158 | (108-213) | 611 | (501-684) | 167 | (154-192) | 6.2 | (5.4-6.9) |
| ***Pirozhky*** | 4 | 100 | (91-121) | 366 | (107-593) | 165 | (115-221) | 3.6 | (1.6-5.8) |
| ***Pirozhnoe* (cake)** | 2 | 102 | (91-113) | 273 | (263-283) | 164 | (120-209) | 3.0 | (2.3-3.7) |
| **Pizza** | 1 | 174 | (174-174) | 609 | (609-609) | 217 | (217-217) | 4.7 | (4.7-4.7) |
| **Salad (cabbage)** | 2 | 101 | (99-104) | 782 | (656-909) | 224 | (206-243) | 5.9 | (5.4-6.3) |
| ***Samsa*** | 1 | 110 | (110-110) | 443 | (443-443) | 130 | (130-130) | 5.8 | (5.8-5.8) |
| **Sausage roll** | 3 | 84 | (71-98) | 433 | (295-604) | 166 | (134-223) | 4.7 | (3.0-7.2) |
| **Soup (lentil)** | 1 | 382 | (382-382) | 311 | (311-311) | 182 | (182-182) | 2.9 | (2.9-2.9) |
| **Wafers** | 3 | 88 | (69-102) | 121 | (75-164) | 122 | (58-177) | 1.8 | (1.6-2.2) |
| K, potassium. Na, sodium. Na/K sodium-potassium ratio | | | | | | | | | |
